# Parametrizing reaction probabilities for proton transfers in protic ionic liquids

**DOI:** 10.3389/fchem.2026.1679806

**Published:** 2026-03-11

**Authors:** Márta Gődény, Adriel Palmisano, Christian Schröder

**Affiliations:** 1 Institute of Computational Biological Chemistry, Faculty of Chemistry, University of Vienna, Vienna, Austria; 2 Vienna Doctoral School in Chemistry, Faculty of Chemistry, University of Vienna, Vienna, Austria

**Keywords:** carboxylates, imidazolium, ionic liquid, proton transfer, quantum mechanics, relaxed scan

## Abstract

Protic ionic liquids are promising electrolytes for electrochemical applications owing to their intrinsic proton conductivity, but quantitative understanding of the underlying proton transfer processes remains limited. Here, we present a systematic quantum-mechanical investigation of proton transfer in a series of 1-methylimidazolium carboxylates, with the specific goal of parametrizing reaction probabilities for use in reactive molecular dynamics simulations. Density functional theory scans were performed to map relaxed potential energy surfaces along two collective variables, the donor–acceptor distance and the proton transfer coordinate. The resulting energy profiles were accurately represented by Morse potentials. From the donor–acceptor distance scans, the distance-dependent reaction probabilities were fitted using a hyperbolic tangent function. Analysis of the proton transfer coordinate revealed small or even negligible energy barriers for the proton transfer reactions, which in turn resulted in low empirical valence bond coupling energies between the reactant and product states. Quantum tunneling effects appear to play only a minor role in these processes. Consequently, the proton transfer reaction probabilities are predominantly governed by thermally activated hopping events, which are captured within a classical kinetic model framework.

## 1 Introduction

Protic ionic liquids (PILs) (Bailey et al., 2024; Belieres and Angell, 2007; Greaves and Drummond, 2008; Yoshizawa et al., 2003; You et al., 2024) represent a distinct subclass of ionic liquids with considerable promise for advanced electrochemical applications. Their inherent properties, including low volatility, high thermal stability, and elevated ionic conductivity, make them attractive for applications in fuel cells, supercapacitors, and solid-state batteries (Bailey et al., 2024; Greaves and Drummond, 2008). Realizing their full technological potential, however, requires a detailed molecular-level understanding of the mechanisms governing hydrogen bonding (Hayes et al., 2013) and the subsequent proton transport (Agmon, 1995), particularly how these mechanisms are modulated by molecular structure and external conditions (Miran et al., 2019). Among the key electrochemical parameters, ionic conductivity is critically influenced by both the concentration of mobile charge carriers and their mobility. In addition, proton hopping can substantially enhance charge transport efficiency in PILs.

PILs are typically generated through proton transfer reactions between Brønsted acids and bases. However, these reactions are often incomplete, and the equilibrium established between the charged and neutral species determines the degree of ionization, leading to a non-stoichiometric composition (You et al., 2024). In the case of common 1-methylimidazolium ( ${\text{Im}}_{1}{\text{H}}^{+}$ ) based PILs this equilibrium reads:

$${\text{Im}}_{1}{\text{H}}^{+}+{\text{Y}}^{-}⇌{\text{Im}}_{1}+\text{HY}$$ (1)

$$K=\frac{\left[{\text{Im}}_{1}\right]\cdot \left[\text{HY}\right]}{\left[{\text{Im}}_{1}{\text{H}}^{+}\right]\cdot \left[{\text{Y}}^{-}\right]}$$ (2)

The equilibrium position is governed by the equilibrium constant K , which is defined as the ratio of the product concentrations to those of the reactants. A large value of K corresponds to a low ionic content, whereas a small value of K indicates a high concentration of ions. Thus, K serves as a measure of the ionicity. To approximate the actual value of K , one typically considers the difference in $pK_{a}$ values (You et al., 2024) that quantifies the acidity difference between the acidic cation ${\text{Im}}_{1}{\text{H}}^{+}$ and the conjugate acid of the base HY (Belieres and Angell, 2007; Doi et al., 2013; Greaves and Drummond, 2008; Yoshizawa et al., 2003). A difference $\Delta pK_{a}=pK_{a}\left({\text{Im}}_{1}{\text{H}}^{+}\right)-pK_{a}\left(\text{HY}\right)$ greater than 6–10 is generally considered sufficient (Greaves and Drummond, 2008; Yoshizawa et al., 2003) to ensure complete proton transfer and, consequently, full ionicity, resulting in a well-defined ionic character of the molecules. However, also much higher $\Delta pK_{a}$ are suggested (Miran et al., 2019). In contrast, smaller $\Delta pK_{a}$ values indicate only partial proton transfer (Stoimenovski et al., 2010), typically leading to the coexistence of ionic and neutral species (Doi et al., 2013; You et al., 2024). However, the individual $pK_{a}$ values are usually only tabulated for aqueous solutions and may not be directly applicable to pure protic ionic liquids. Only for some imidazolium-based ionic liquids spectrophotometric methods based on the Hammett function exist to determine the acidity of particular molecules (Thomazeau et al., 2003; Robert et al., 2009; D’Anna et al., 2009; Zhang et al., 2016; Rensonnet and Malherbe, 2024; Rensonnet et al., 2025).

**FIGURE 1:**
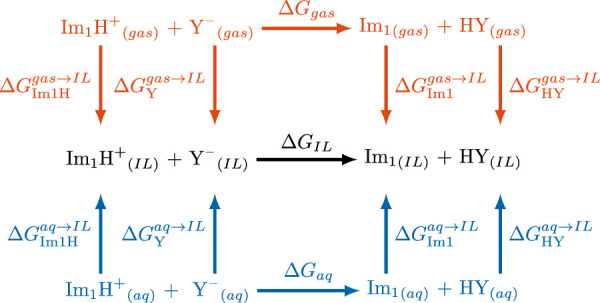
Thermodynamic cycles of the acid/base reaction ${\text{Im}}_{1}{\text{H}}^{+}+{\text{Y}}^{-}⇌{\text{Im}}_{1}+\text{HY}$ in gas, ionic liquid and aqueous phase (Zheng et al., 2024).

From a theoretical point of view, the equilibrium constant K is a function of the solvent and consequently the free reaction energy ΔG :

ΔG=−RT⁡ln⁡K (3)

varies between the gas phase, ionic liquids, and aqueous solutions. Thermodynamic cycles can be employed to determine $\Delta G_{IL}$ either from aqueous-phase data (typically employed to adjust or correlate with experimental observations (Ghalami-Choobar and GHiami-Shomami, 2013; Ribeiro Dutra et al., 2021; Zheng et al., 2024), blue path in Figure 1 and Equation 4) or from gas-phase calculations (commonly used in quantum-mechanical (QM) studies (Busch et al., 2022; Fujiki et al., 2021; Zheng et al., 2024), orange path in Figure 1 and Equation 5):

$$\Delta G_{IL}=\Delta G_{aq}+\left(\Delta G_{\text{Im}1}^{aq\to IL}+\Delta G_{\text{HY}}^{aq\to IL}\right)-\left(\Delta G_{\text{Im}1\text{H}}^{aq\to IL}+\Delta G_{\text{Y}}^{aq\to IL}\right)=\Delta G_{aq}+\delta \Delta G^{aq\to IL}$$ (4)

$$=\Delta G_{\mathrm{gas}}+\left(\Delta G_{\text{Im}1}^{\mathrm{gas\to IL}}+\Delta G_{\text{HY}}^{\mathrm{gas\to IL}}\right)-\left(\Delta G_{\text{Im}1\text{H}}^{\mathrm{gas\to IL}}+\Delta G_{\text{Y}}^{\mathrm{gas\to IL}}\right)=\Delta G_{\mathrm{gas}}+\delta \Delta G^{\mathrm{gas\to IL}}$$ (5)

Since $\Delta G_{aq}=2.303RT\cdot \Delta pK_{a}$ corresponds to the Gibbs free energy derived from $\Delta pK_{a}$ values, the large discrepancies (Miran et al., 2019) in the upper limit for complete ionicity may arise from $\delta \Delta G^{aq\to IL}$ characterizing the situation in the actual ionic liquid instead of the aqueous phase. For example, carbon acids are stronger acids in water than in 1-methylimidazolium-based ionic liquids. Nevertheless, the equilibrium described in Equation 1 lies on the right-hand side in the pure protic ionic liquid, demonstrating the pronounced effect of $\delta \Delta G^{aq\to IL}$ . Gibbs free energies are associated with the formation or modification of molecular cavities, including changes in hydrogen bonding, electrostatic interactions and dispersion contributions within the different solvation media. For example, the dielectric constant of ionic liquids is typically about one-fifth that of water, indicating significantly weaker ion solvation compared to aqueous environments. Consequently, the equilibrium constant K in Equation 2 may be higher in ionic liquids than predicted from $\Delta pK_{a}$ , leading to a shift of ionicity in pure protic ionic liquids toward lower values. Since fully explicit solvation is computationally prohibitive in QM calculations, polarizable continuum models offer a practical alternative in this respect (Fujiki et al., 2021).

Partial ionicity x or non-stoichiometry (You et al., 2024) can compromise key IL properties, including phase stability and volatility. Nonetheless, these pseudo-ionic systems can still exhibit substantial ionic conductivities (Chen et al., 2014; Greaves and Drummond, 2008; Gődény et al., 2024; Hou et al., 2011; Jacobi et al., 2022; MacFarlane et al., 2006; Thawarkar et al., 2019). Quantitative characterization of the ionic versus neutral composition of PILs remains challenging due to inconsistencies among experimental techniques: NMR studies (Calandra et al., 2024; Chen et al., 2018; Hasani et al., 2019; Hoarfrost et al., 2012; Kobrak et al., 2025; Overbeck and Ludwig, 2018), quasi-elastic neutron scattering (Hoarfrost et al., 2012), small-angle X-ray scattering (Calandra et al., 2024; Kobrak et al., 2025), Raman (Doi et al., 2013; Watanabe et al., 2022) and IR spectroscopy (Kobrak et al., 2025; Mann et al., 2020; Sun et al., 2016) have yielded conflicting results. For example, Kanzaki et al. (2008), Kanzaki et al. (2010), and Song et al. (2012) employed potentiometric titration to study the proton activity of several PILs and found that, in contrast to conventional systems such as ethylammonium nitrate, the 1-methylimidazole ( ${\text{Im}}_{1}$ ) plus acetic acid mixture exhibited no significant inflection point in the titration curve. From calorimetric titration data, the authors inferred a negative $pK_{a}$ of approximately −1.4, corresponding to a charged species fraction of x = 33.6%. Subsequent density-based analyses by Qian et al. (2012) and Hou et al. (2011) yielded comparable estimates, with charged fractions x ranging from 31.4 % to 37.3%. However, subsequent Raman spectroscopy by the same group proposed an negligible charged species content of x = 1.0% (Doi et al., 2013). In contrast, Chen et al. (2018) reported a much higher charged fraction x of 92.8% based on NMR spectroscopy. Assuming the absence of any excess neutral species ${\text{Im}}_{1}$ and HY , the equilibrium constant K in Equation 2 can be approximated in Equation 6 as:

$$K≃\frac{{\left(1-x\right)}^{2}}{x^{2}}$$ (6)

$$x=\frac{\left[{\text{Im}}_{1}{\text{H}}^{+}\right]}{\left[{\text{Im}}_{1}{\text{H}}^{+}\right]+\left[{\text{Im}}_{1}\right]}=\frac{\left[{\text{Y}}^{-}\right]}{\left[{\text{Y}}^{-}\right]+\left[\text{HY}\right]}$$ (7)

in terms of the partial ionicity x , which depends on the respective concentrations of the charged and neutral species in Equation 7. Based on the previously reported ionicity, the corresponding Gibbs free reaction energy $\Delta G_{IL}$ (see Equation 3) is approximately −3.5 kJ mol^−1^ for a system exhibiting about 30% ionic character. When adopting the high ionicity reported by Chen et al. (2018), the free reaction energy increases to +12.7 kJ mol^−1^. Despite these significant discrepancies, there is a general consensus that the 1-methylimidazole ( ${\text{Im}}_{1}$ )/acetic acid system exhibits limited ionic character and thus cannot be considered a fully ionic liquid.

The above mentioned experimental techniques are often capable of probing the overall acid–base equilibria in PILs, but they fail in resolving mechanistic details. In particular, side reactions (Jacobi et al., 2022; You et al., 2024) such as:

$$\text{HY}+{\text{Y}}^{-}⇌{\text{Y}}^{-}+\text{HY}$$ (8)

$${\text{Im}}_{1}{\text{H}}^{+}+{\text{Im}}_{1}⇌{\text{Im}}_{1}+{\text{Im}}_{1}{\text{H}}^{+}$$ (9)

do not affect the thermodynamic position of the acid–base equilibrium and are therefore of limited interest from a classical chemical standpoint. Nonetheless, these processes can enhance ionic conductivity by enabling transient proton transfer via hopping mechanisms (Agmon, 1995). Moreover, most experimental approaches lack the spatial and temporal resolution required to unravel the stepwise dynamics of proton hopping at the molecular level. While density functional theory (DFT) calculations can provide valuable insights into the potential energy surface, this static perspective remains insufficient for a comprehensive understanding of dynamic transport phenomena (Gődény and Schröder, 2025). Ab initio molecular dynamics (AIMD) simulations offer a more realistic, time-resolved picture of proton transfer mechanisms (Brehm et al., 2012; Ingenmey et al., 2018), but their system sizes and simulation periods are too small to compute macroscopic properties such as the conductivity. Nevertheless, AIMD remains a powerful tool for elucidating the fundamental steps of proton transport. To overcome these limitations, reactive molecular dynamics methods such as ReaxFF (Senftle et al., 2016) have been developed. ReaxFF replaces the QM description with an empirical bond-order-dependent force field, allowing for simulation of bond breaking and formation over longer timescales. Despite its flexibility, ReaxFF suffers from a complex and system-specific parametrization procedure, which often lacks generalizability. An alternative and more promising approach is provided by empirical valence bond (EVB) methods (Arntsen et al., 2016; Day et al., 2002; Kamerlin and Warshel, 2010; Knight et al., 2012; Stoppelman and McDaniel, 2020; Wu et al., 2008; Čuma et al., 2000), which have been successfully employed to model proton transport in aqueous systems (Schmitt and Voth, 1998; Schmitt and Voth, 1999) and in mixtures of aprotic ionic liquids with water (Stoppelman and McDaniel, 2020). However, to keep the computational effort in these simulations reasonable, only a small region in the simulation box is reactive and the rest of the box is described by conventional non-reactive force fields.

In our in-house simulation framework PROTEX (Gődény et al., 2024; Gődény and Schröder, 2025; Joerg et al., 2023), the entire simulation box is treated as reactive, enabling large-scale simulations of proton dynamics, but the method does not explicitly resolve the full proton transfer pathway on the atomic scale. Instead, transitions between reactant and product states of hydrogen-bonded donor–acceptor pairs are represented by forward and backward reaction probabilities (Jacobi et al., 2022), which include not only primary proton transfer in Equation 1 but also the side reactions in Equations 8, 9. The computational overhead introduced by this reactive scheme is modest, typically on the order of 10%–20% relative to a polarizable, non-reactive MD simulation, depending on the frequency at which proton transfer events are evaluated. This computational efficiency allows for simulations of sufficiently large systems and time scales to calculate transport properties such as ionic conductivity. For example, in the case of 1-methylimidazolium acetate $\left[{\text{Im}}_{1}\text{H}\right]{\text{CH}}_{3}\text{COO}$ , the conductivity obtained from PROTEX simulations (Joerg et al., 2023) was in closer agreement with experimental values than that from non-reactive, polarizable MD simulations, even when the ionicity in the latter was adjusted to match experimental density, diffusion coefficients, and the dielectric response of the PIL. Nevertheless, both PROTEX and EVB simulations require extensive QM potential energy surface scans for parametrization. In the present work, we focus on such QM scans for a series of PILs based on 1-methylimidazolium paired with formate, acetate, propanoate, and butanoate anions. From these scans, we aim to elucidate the microscopic mechanisms of proton transfer. Our analysis emphasizes the possible formation of short, strong hydrogen bonds between the Brønsted acid and base, exemplified by $[{\text{Im}}_{1}\text{H}{]}^{+}\cdots {\text{Y}}^{-}$ , which may stabilize a partially delocalized proton. Such species are often associated with a double-well potential energy surface, in which the proton may undergo thermally activated or quantum-assisted migration. A detailed understanding of this potential energy landscape can provide mechanistic insights into the proton transfer process and clarify the conditions under which delocalization, tunneling, or classical over-the-barrier transfer dominate.

## 2 Methods

**FIGURE 2:**
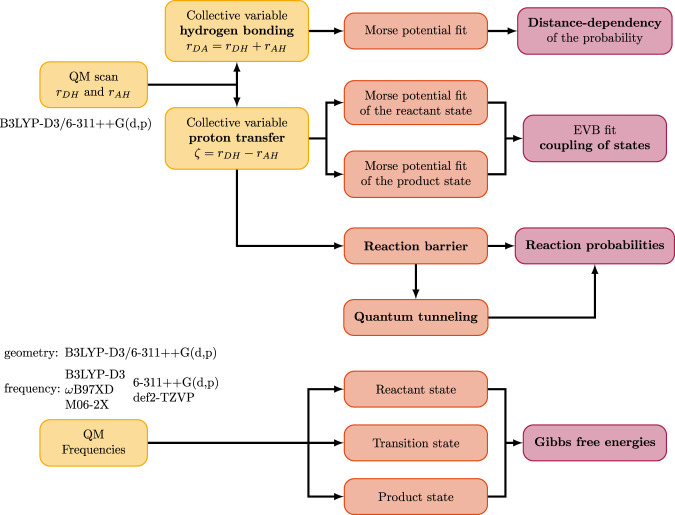
Workflow of the analysis of the QM scans and frequency calculations in this work.

All QM calculations were performed using the GAUSSIAN09 software package (Frisch et al., 2016). We tested the following functional/basis set combinations: B3LYP-D3/6-311++G(d,p), B3LYP-D3/def2-TZVP, ω B97XD/6-311++G(d,p), and M06-2X/6-311++G(d,p). The B3LYP-based calculations additionally included Grimme’s D3 dispersion correction. Solvent effects were accounted for using a polarizable continuum model with a dielectric constant of 15, which approximately represents the dielectric environment of PILs. For each system, multiple initial geometries of the hydrogen-bonded acid–base complex were subjected to full geometry optimization. The lowest-energy structure, corresponding to the global minimum on the potential energy surface, was subsequently used as the starting point for various relaxed potential energy scans: The proton transfer reaction can be characterized by monitoring the collective variable associated with hydrogen bonding, which reflects the variation in donor–acceptor distance. Analysis of this coordinate reveals the distance dependence of the reaction probability. Alternatively, forward and backward scans of the donor–hydrogen (or acceptor–hydrogen, in the reverse direction) distance provide a suitable collective variable for describing the proton transfer process. These investigations enable the construction of an EVB model that captures the coupling between the relevant states. From the barrier height obtained in this framework, reaction probabilities can be estimated using a kinetic model. Moreover, the shape of the reaction barrier governs the extent of quantum tunneling contributions. The overall workflow of our analysis is illustrated in Figure 2, and the individual steps are detailed in the following sections.

### 2.1 Collective variable concerning the hydrogen bonding

**FIGURE 3:**
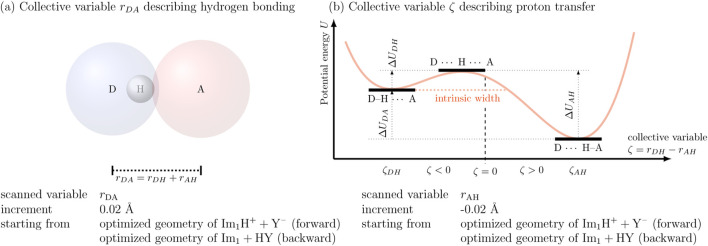
Collective variables describing the proton transfer reaction in PILs: **(a)** The distance $r_{DA}$ quantifies the hydrogen bond between the donor (D) and acceptor (A) atoms. **(b)** The difference $\zeta =r_{DH}-r_{AH}$ characterizes the proton transfer coordinate, with the transition state located near ζ=0 . Increasing $r_{DA}$ moves $\zeta_{DH}$ to the left and $\zeta_{AH}$ to the right.

Lill and Helms (2001a) introduced a collective variable $r_{DA}$ to characterize hydrogen bonding, defined as:

$$r_{DA}=r_{DH}+r_{AH}$$ (10)

that is, the sum of the donor–hydrogen $r_{DH}$ and acceptor–hydrogen $r_{AH}$ distances. This relation holds strictly only for linear configurations. As illustrated in Figure 3a, the Lennard-Jones spheres of the donor and acceptor atoms significantly overlap in the optimized geometry of the hydrogen-bonded donor–acceptor pair, placing the system in the repulsive regime of the Lennard-Jones potential. This steric repulsion is counterbalanced by the strong electrostatic attraction between the proton and the acceptor atom A. Typically, the Lennard-Jones sphere of the hydrogen lies entirely within that of the donor atom D.

#### 2.1.1 Relaxed forward and backward scans

For each 1-methylimidazolium carboxylate system studied, we conducted independent relaxed potential energy scans for both the ion pair $[{\text{Im}}_{1}\text{H}{]}^{+}+{\text{Y}}^{-}$ and the neutral pair $\text{HY}+{\text{Im}}_{1}$ , representing the reactant and product states of the acid–base equilibrium in Equation 1. Starting from the fully optimized minimum-energy structure, the donor–acceptor distance $r_{DA}$ (instead of the individual $r_{DH}$ and $r_{AH}$ in Equation 10) was incrementally increased in steps of 0.02 Å. At each increment, constrained geometry optimizations were performed in which $r_{DA}$ was held fixed while allowing all other degrees of freedom to relax. The resulting energies define a one-dimensional relaxed potential energy profile along the proton transfer coordinate. To avoid premature or artificial proton transfer during the scans, the lower bound of $r_{DA}$ for the $[{\text{Im}}_{1}\text{H}{]}^{+}\cdots {\text{Y}}^{-}$ configuration was set to 2.5 Å, as smaller values led to spontaneous transfer of the proton to the acceptor. For donor–acceptor distances exceeding 3.2 Å, structural instabilities were observed: the geometry reorganized such that the second oxygen atom of the carboxylate group became closer to the donor site than the original acceptor oygen. This configurational rearrangement compromised the validity of the scan, and thus the analysis was restricted to the distance range within which the hydrogen bond geometry remained stable and chemically meaningful.

#### 2.1.2 Fitting using Morse potentials

While Lill and Helms successfully employed quadratic functions to model distance-dependent potential energy profiles in systems such as ammonium and water (Lill and Helms, 2001a; Lill and Helms, 2001b; Lill and Helms, 2001c), we found this approach insufficient for describing the energetics of proton transfer in PIL systems. Even near equilibrium geometries, the energy landscapes of these hydrogen-bonded complexes exhibited significant anharmonicity, rendering quadratic fits inadequate. To address this, we adopted a Morse potential to represent the energy profiles more accurately:

$$U^{{\text{Im}}_{1}{\text{H}}^{+}\cdots {\text{Y}}^{-}}\left(r_{DA}\right)≃D{\left(1-\exp\left[-\alpha \left(r_{DA}-r_{DA}^{0}\right)\right]\right)}^{2}$$ (11)

$$U^{\text{HY}\cdots {\text{Im}}_{1}}\left(r_{DA}\right)≃D{\left(1-\exp\left[-\alpha \left(r_{DA}-r_{DA}^{0}\right)\right]\right)}^{2}$$ (12)

$$\Delta U_{DA}\left(r_{DA}\right)=U^{\text{HY}\cdots {\text{Im}}_{1}}\left(r_{DA}\right)-U^{{\text{Im}}_{1}{\text{H}}^{+}\cdots {\text{Y}}^{-}}\left(r_{DA}\right)$$ (13)

$$≃D{\left(1-\exp\left[-\alpha \left(r_{DA}-r_{DA}^{0}\right)\right]\right)}^{2}+D_{0}$$ (14)

Here, D denotes the dissociation energy, $r_{DA}^{0}$ the equilibrium donor–acceptor distance, and α a parameter related to the potential width. The energy difference $\Delta U_{DA}\left(r_{DA}\right)$ in Equation 13 corresponds to the term $E_{12}$ as defined in the original work by Lill and Helms (2001a), Lill and Helms (2001b), and Lill and Helms (2001c). Since the individual potential energy profiles for the protonated and deprotonated complexes are now represented by Morse potentials, their difference $\Delta U_{DA}\left(r_{DA}\right)$ is also more accurately modeled by a Morse-type expression, as shown in Equation 14.

#### 2.1.3 Side reactions

In principle, the energy differences associated with the side reactions given in Equations 8, 9 should vanish, due to the symmetry inherent in proton exchange: the acceptor in one configuration becomes the donor in the other after proton transfer, suggesting energetic equivalence between the two complexes. However, in practice, the mutual orientation of the donor–acceptor pair differs due to the inherent asymmetry of the hydrogen-bonded complex resulting in non-negligible energy discrepancies between the nominally equivalent configurations. Unfortunately, attempts to generate relaxed potential energy scans for the $\text{HY}\cdots {\text{Y}}^{-}$ complex were unsuccessful. During the constrained optimization procedure, the system underwent spontaneous reorientation: the carboxylate anion rotated, positioning its second oxygen atom closer to the hydroxyl oxygen, thereby disrupting the intended donor–acceptor geometry. Similar issues were encountered when the scan was defined using the positions of the carboxylate carbon atoms as reference points. In both cases, the system relaxed into an alternative local minimum, precluding a meaningful scan of the intended proton transfer pathway and rendering these data unsuitable for further analysis.

### 2.2 Collective variable concerning the proton transfer

Two-dimensional potential energy surface scans of the hydrogen-bonded donor–acceptor complexes revealed that proton transfer occurs predominantly along a direct, co-linear pathway between the donor and acceptor atoms (Jacobi et al., 2022). This observation justifies the use of one-dimensional reaction coordinate scans to capture the essential features of the proton transfer process, including the identification of the transition state and the associated energy barrier. To describe the position of the proton along this transfer path, we define a second collective variable ζ as the difference in bond lengths between the proton and the donor and acceptor atoms:

$$\zeta =r_{DH}-r_{AH}$$ (15)

This coordinate provides a continuous measure of the proton’s location relative to the two binding sites. According to the Hammond postulate, the transition state generally lies closer to the less stable (higher-energy) species, either the reactant or product as indicated by the dashed line in Figure 3b. As such, the transition state does not necessarily occur at ζ=0 , although this is often used as a reference. In our relaxed potential energy scans, however, the transition state consistently occurred near ζ=0 , indicating a nearly symmetric proton-sharing geometry at the energy barrier. Negative values of ζ imply that the proton remains more strongly associated with the donor atom, corresponding to the reactant-like configuration, whereas positive ζ values indicate that the proton is more localized on the acceptor, representing the product-like state.

#### 2.2.1 Relaxed forward and backward scans

To map the potential energy landscape along the proton transfer coordinate ζ , we performed relaxed scans by incrementally decreasing the hydrogen–acceptor distance $r_{AH}$ in steps of 0.02 Å, beginning from the optimized minimum-energy geometries of both the ion pair ${\text{Im}}_{1}{\text{H}}^{+}\cdots {\text{Y}}^{-}$ and the neutral complex $\text{HY}\cdots {\text{Im}}_{1}$ . At each step, the geometry was optimized under the constraint of a fixed $r_{AH}$ , while all remaining degrees of freedom were allowed to relax. For each configuration along the scan, we recorded $r_{DH}$ and $r_{AH}$ and the corresponding energy to facilitate the calculation of ζ according to Equation 15. Although the scans were intended to follow a one-dimensional reaction coordinate, the donor–acceptor distance $r_{DA}$ was not held constant and exhibited slight variations as a consequence of geometric relaxation. A representative energy profile along ζ is presented in Figure 3b. The position of the transition state, characterized by a near-symmetric D⋯H⋯A configuration, was further validated through explicit transition state optimizations. The intrinsic width (Qiu and Schreiner, 2023) of the energy barrier is the distance from ζ of the state with the higher energy (in Figure 3b the reactant state) to the ζ where $U\left(\zeta \right)=U\left(\zeta_{\text{DH}}\right)$ closest to $\zeta_{\text{AH}}$ . Smaller intrinsic barrier width indicate higher chances for tunneling and vice versa.

The forward activation barrier $\Delta U_{DH}$ was defined as the energy difference between the transition state and the reactant state (with the proton initially bound to the donor), while the reverse barrier $\Delta U_{AH}$ corresponds to the energy difference between the transition state and the product state (with the proton transferred to the acceptor). The overall energy difference between the reactant and product states is then given by $\Delta U_{DA}=\Delta U_{AH}-\Delta U_{DH}$ . Nevertheless, as anticipated from the analysis of the first collective variable $r_{DA}$ (see Equation 14), the forward and reverse barrier heights can differ significantly. This asymmetry in activation energies has been previously reported for 1-methylimidazolium acetate (Jacobi et al., 2022) and can be attributed to the inherent thermodynamic imbalance between the ionic and neutral species. Since the neutral complexes are generally more stable than their ionic counterparts, the forward proton transfer in Equation 1 is typically associated with a lower activation energy than the reverse reaction.

### 2.3 Computing Gibbs free energies

So far, only potential energy profiles U(ζ) have been discussed, while the Gibbs free reaction energy $\Delta G_{IL}$ was related to the equilibrium constant K of the proton transfer reaction in the Introduction. The latter include additional contributions arising from zero-point energies ΔZPE , thermal corrections $\Delta H_{\text{Thermal}}$ , and entropic effects, which are essential for obtaining a realistic thermodynamic description of proton transfer processes. It can be approximated from QM calculations as:

$$\Delta G_{IL}≃\Delta U_{DA}+\Delta ZPE+\Delta H_{\text{Thermal}}-T\Delta S$$ (16)

using partition functions for the last two contributions. The accurate determination of $\Delta G_{IL}$ values in Equation 16 hence requires frequency calculations, which cannot be performed along a potential energy scan but only for selected configurations such as reactant, transition, and product state. The GAUSSIAN09 calculations employed the additional options SCF = (XQC,VeryTight) and Integral = UltraFine to ensure reliable self-consistent field convergence and improved numerical accuracy of the low vibrational frequencies, which are essential for the evaluation of Gibbs free energies.

#### 2.3.1 Free energy correction by GoodVibes

Frequency analyses are commonly performed within the framework of the rigid-rotor harmonic-oscillator approximation (Conquest et al., 2021; Maehr et al., 2025; Pracht and Grimme, 2021). However, this model fails to provide meaningful partition functions for low-frequency modes because such motions are intrinsically flat and strongly anharmonic (East and Radom, 1997; Pracht and Grimme, 2021; Velmiskina et al., 2025), particularly in the case of torsional and intermolecular rotational degrees of freedom. As a result, this approximation systematically overestimates the accessible phase space and, consequently, the entropic contribution. A more physically accurate description requires the use of hindered- or free-rotor models that smoothly interpolate between the harmonic and free-rotor limits (East and Radom, 1997). Moreover, the rigid-rotor harmonic-oscillator treatment is typically based on a single optimized geometry and therefore neglects the influence of multiple thermally accessible conformers, whose ensemble contributions can substantially affect the total entropy (Pracht and Grimme, 2021; Suárez et al., 2011). Because entropic terms scale inversely with vibrational frequency, inaccuracies in the low-frequency region dominate, leading to unphysically large entropy values for these modes (Pracht and Grimme, 2021; Velmiskina et al., 2025).

The quasi-harmonic correction introduced by Grimme and implemented in GoodVibes (Luchini et al., 2020) partially mitigates these issues by damping the entropic contributions of soft vibrational modes. Nevertheless, this approach still assumes a well-defined stationary point on the potential-energy surface for the evaluation of normal-mode frequencies. In practice, many transition states, particularly those associated with barrierless or diffusion-limited proton transfer events, do not correspond to sharp first-order saddle points, as we have observed for all investigated carboxylates except formate. In such cases, the Hessian matrix becomes extremely sensitive to small geometric variations, and small coordinate changes can drastically alter the low-frequency spectrum. Consequently, the computed Gibbs free energy of the transition state may spuriously fall below that of the reactant or product, despite its higher potential energy, as observed for the reaction ${\text{Im}}_{1}{\text{H}}^{+}+{\text{Y}}^{-}⇌{\text{Im}}_{1}+\text{HY}$ . This artifact originates from an overestimation of vibrational entropy and underscores the breakdown of both the rigid-rotor and quasi-harmonic approximations for systems exhibiting strongly anharmonic, large-amplitude motions (Dzib et al., 2024). As a consequence, the reaction barriers for both the forward and reverse processes in this work were evaluated from the potential energy differences between the reactant or product states and the corresponding transition state.

## 3 Results and discussion

### 3.1 Comparison to experimental results

#### 3.1.1 Selection of the functional and basis set

For the QM potential energy scans, we employed the following functional/basis set combinations: B3LYP-D3/6-311++G(d,p), B3LYP-D3/def2-TZVP, ω B97XD/6-311++G(d,p), and M06-2X/6-311++G(d,p). The B3LYP-based calculations additionally included Grimme’s D3 dispersion correction. Omitting the Grimme dispersion correction, or employing functionals (such as MP2) for which such a correction is unavailable, leads to substantially larger energy differences between the reactant and product states. All QM calculations were performed within a polarizable continuum model using a dielectric constant of ϵ=15 , representative of typical ionic liquids. Vibrational frequency analyses were conducted for the reactant, transition, and product states to confirm their nature and to compute Gibbs free energies. These were evaluated using GoodVibes (Luchini et al., 2020), incorporating zero-point energy as well as entropic contributions. The geometries of the reactant and product species were obtained from full geometry optimizations at the B3LYP-D3/6-311++G(d,p) level of theory. Except for 1-methylimidazolium formate ( $\tilde{\nu }=$ −887 cm^−1^), the transition states exhibited very shallow potential energy surfaces, still characterized by single imaginary frequencies below 100 cm^−1^, which were highly sensitive to the optimization parameters. Therefore, our analysis focused on the Gibbs free energy differences between the reactant and product states.

**FIGURE 4:**
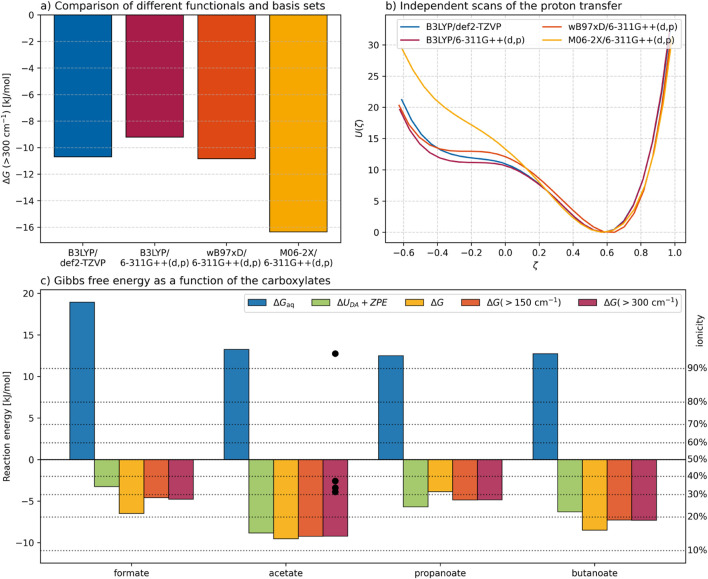
Comparison of reaction energies: **(a)** Gibbs free energy change of various functionals and basis sets for the reaction ${\text{Im}}_{1}{\text{H}}^{+}+{\text{CH}}_{3}{\text{COO}}^{-}⇌{\text{Im}}_{1}+{\text{CH}}_{3}\text{COOH}$ , **(b)** corresponding potential energy scan for the same reaction, **(c)** Gibbs free energy difference as a function of the carboxylates. The comparison to experiment can only be performed for 1-methylimidazolium acetate (black dots). However, the prediction based on $\Delta pK_{a}$ yields very positive ΔG in contradiction to the experimental found ionicities.

For the tested functional and basis set combinations, the calculated Gibbs free energy differences ranged from −9.2 kJ mol^−1^ to −16.3 kJ mol^−1^, as shown in Figure 4a. This variation is relatively minor compared to discrepancies typically observed among different density functionals (Brémond et al., 2022). Given that the experimental ionicities are approximately −3.5 kJ mol^−1^, the B3LYP-D3/6-311++G(d,p) combination, including diffuse and polarization functions as well as Grimme’s dispersion correction, yielded the best agreement with experiment, whereas the M06-2X/6-311++G(d,p) results deviated most strongly. Additionally, independent potential energy scans were performed for the reaction ${\text{Im}}_{1}{\text{H}}^{+}+{\text{Y}}^{-}⇌{\text{Im}}_{1}+\text{HY}$ as depicted in Figure 4b, using the same color code as in Figure 4a. The potential minimum at ζ=−0.6 corresponds to the neutral complex between 1-methylimidazole and acetic acid and is consistently reproduced by all methods. Likewise, the repulsive region at more negative ζ values shows good agreement across all QM calculations. In contrast, the B3LYP-D3/def2-TZVP and M06-2X/6-311++G(d,p) scans did not identify a stable configuration corresponding to 1-methylimidazolium acetate. The local minimum obtained with ω B97XD/6-311++G(d,p) was approximately 2 kJ mol^−1^ higher in energy than that from B3LYP-D3/6-311++G(d,p). Based on these results, B3LYP-D3/6-311++G(d,p) was selected for all subsequent calculations as it is closest to experimental data.

#### 3.1.2 Gibbs free energies and ionicity

The frequency calculations for the reactant, transition, and product states of the investigated carboxylate systems enabled the inclusion of zero-point energy corrections to the potential energy. The resulting values of $\Delta U_{\text{DA}}+\Delta ZPE$ are shown as green bars in Figure 4c. The uncorrected Gibbs free energies ΔG obtained directly from Gaussian output are represented by yellow bars and deviate on average by approximately 2 kJ mol^−1^ from the corresponding potential energy values. Applying GoodVibes (Luchini et al., 2020) corrections using frequency thresholds of 150 cm^−1^ (orange bars) and 300 cm^−1^ (red bars) significantly modifies the free energy values, bringing them into closer agreement with the ZPE-corrected potential energies. The similarity between the two GoodVibes-corrected datasets also indicates that the correction procedure has converged.

The computed Gibbs free energies can be compared to $\Delta G_{IL}$ values. For 1-methylimidazolium acetate, $\Delta G_{IL}$ is −3.5 kJ mol^−1^, derived from the experimentally reported ionicity by Kanzaki et al. (2008), Kanzaki et al. (2010), and Song et al. (2012) (black dots in Figure 4c, corresponding to approximately one-third ionized species. In contrast, the QM derived ΔG of −9.2 kJ mol^−1^ suggests a lower effective ionicity. For the remaining carboxylates, calculated values between −4 kJ/mol to −7 kJ/mol imply ionicity levels of roughly 20%–30%, consistent with expectations for these PILs. It should be emphasized that these QM results are based on single configurations of the reactant and product states for a single molecular pair. Moreover, the standard density functional B3LYP and a polarizable continuum model were employed to maintain computational feasibility.

The present QM analysis does not explicitly account for the concentration-dependent equilibria of molecular species. In a previous study (Jacobi et al., 2022), we employed a Markov model to evaluate whether QM derived probabilities reproduce the experimentally observed composition. This approach yielded excellent agreement for 1-methylimidazolium acetate, with probabilities comparable to those reported in this work. Furthermore, these probabilities were successfully applied to predict experimental conductivities (Gődény et al., 2024; Joerg et al., 2023). Additional investigations demonstrated that assuming an ionicity of approximately 30% reproduces the experimental density, diffusion coefficients, and dielectric spectra of 1-methylimidazolium acetate (Joerg and Schröder, 2022). Hence, we expect that all sets of reaction probabilities for the 1-methylimidazolium carboxylate systems investigated in this work result in reasonable compositions.

#### 3.1.3 The failure of $pK_{a}$ estimates

Figure 4c also includes Gibbs free energies estimated from $\Delta pK_{a}$ values (blue bars), which are strictly valid only in aqueous solution. These estimates are evidently inadequate in the ionic liquid environment due to the significant contribution of the solvation correction term $\delta \Delta G^{aq\to IL}$ in Equation 4. For 1-methylimidazolium acetate, this correction amounts to approximately −15 kJ mol^−1^, substantially reducing the apparent ionicity and shifting the equilibrium toward a non-stoichiometric composition (You et al., 2024).

In addition to the arguments from the thermodynamics cycles in Figure 1, also in aqueous biological systems with unfavorable $\Delta pK_{a}$ proton transfer reactions may occur because of $pK_{a}$ shifts due to local environment polarity and electrostatics as well as binding interactions at the transition state (Silverstein, 2021).

### 3.2 Analysis of the collective variable concerning hydrogen bonding

**FIGURE 5:**
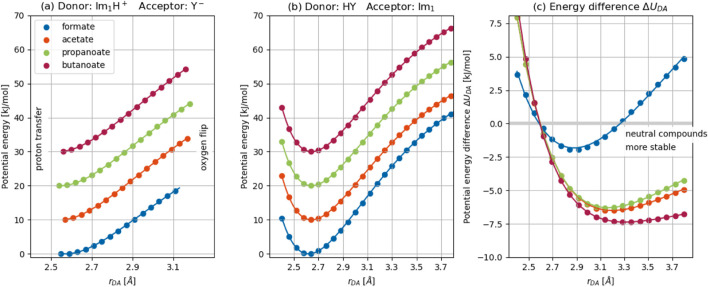
Potential energy profiles for the hydrogen-bonded complexes Im_1_H^+^⋯Y^−^ **(a)** and $\text{H}\text{Y}\cdots {\text{Im}}_{1}$ **(b)** as a function of donor–acceptor distance $r_{DA}$ for a series of 1-methylimidazolium carboxylates. Solid lines correspond to fitted Morse potentials. The resulting energy differences between the complexes are shown in **(c)**. To improve visual clarity, the energy curves for the various 1-methylimidazolium carboxylates have been vertically offset in increments of 10 kJ/mol.

The initial step of the proton transfer reaction involves the formation of a hydrogen bond between the donor and acceptor atoms. The associated potential energy profiles of these hydrogen-bonded complexes as a function of the donor–acceptor distance $r_{DA}$ are presented in Figures 5a,b. To improve visual clarity, the energy curves for the various 1-methylimidazolium carboxylates have been vertically offset in increments of 10 kJ mol^−1^.

#### 3.2.1 Morse potential analysis of hydrogen-bonding

As outlined in the Methods section, the energy profiles were fitted using Morse potentials in Equations 11, 12, and the resulting parameters are summarized in Table 1. Although Morse potentials are traditionally used to model intramolecular bond dissociation, they also provide an excellent description of the intermolecular hydrogen bonds formed in these PIL systems. It becomes evident from the plots that a simple quadratic approximation, such as the parabolic fits originally employed by Lill and Helms, is inadequate for capturing the anharmonicity of the potential energy surface in these systems, particularly beyond the immediate vicinity of the equilibrium geometry. In contrast, the Morse potential offers a more realistic depiction of the energy landscape, accurately reproducing the curvature near the minimum as well as the asymptotic behavior at larger donor–acceptor separations. Moreover, the dissociation energies derived from the Morse potential fits allow for a meaningful quantitative comparison across different anions. It should be noted, however, that the dissociation energy extracted from these fits encompasses not only the hydrogen bond strength but also additional contributions from van der Waals interactions and longer-range electrostatics between the donor and acceptor species.

**TABLE 1:** Parameters of the Morse potential of the scans described in Equations 11, 12.

Anion	${\text{Im}}_{1}{\text{H}}^{+}\cdots {\text{Y}}^{-}$			$\text{HY}\cdots {\text{Im}}_{1}$			$\Delta U_{DA}$			
	D [kJ/mol]	α [Å^−1^]	$r_{DA}^{0}$ [Å]	D [kJ/mol]	α [Å^−1^]	$r_{DA}^{0}$ [Å]	D [kJ/mol]	α [Å^−1^]	$r_{DA}^{0}$ [Å]	$D_{0}$ [kJ/mol]
Formate	44.9	1.92	2.57	56.7	1.65	2.62	19.9	0.92	2.87	−1.8
Acetate	51.6	1.84	2.55	49.8	1.70	2.64	6.5	1.14	3.20	−6.5
Propanoate	50.2	1.87	2.55	49.5	1.71	2.64	8.3	1.10	3.16	−6.3
Butanoate	54.7	1.76	2.54	49.5	1.71	2.64	2.3	1.44	3.30	−7.4

As summarized in Table 1, the behavior of 1-methylimidazolium formate differs substantially from that of the other carboxylate-based PILs. For acetate, propanoate, and butanoate, the fitted Morse parameters are nearly identical, suggesting that the length and nature of the aliphatic chain have minimal influence on the hydrogen bonding interaction with 1-methylimidazole. This is already evident from the unshifted scan data shown in Figures 5a,b. Thus, the observed agreement is not an artifact of the fitting procedure but rather an intrinsic feature of the underlying QM data. This observation is also physically reasonable, since proton transfer processes generally occur on much shorter time scales than internal rotations. Moreover, even for butanoate, the alkyl chain is insufficiently long to interact with the neighboring molecule without disrupting the hydrogen-bonded donor–hydrogen–acceptor geometry. Except for 1-methylimidazolium formate, the dissociation energy of the charged complex ${\text{Im}}_{1}{\text{H}}^{+}\cdots {\text{Y}}^{-}$ slightly exceeds that of the corresponding neutral complex $\text{HY}\cdots {\text{Im}}_{1}$ , indicating a marginally stronger interaction in the ionic form. In addition, the equilibrium donor–acceptor distance for the charged complex is approximately 2.55 Å, which is slightly shorter than the 2.64 Å found for the neutral pair. These $r_{DA}$ values correspond to $r_{NH}$ or $r_{OH}$ distances of approximately 1.6 Å, which are just above the hydrogen bond cutoff adopted in our recent PROTEX simulations (Gődény et al., 2024). The curvature parameter α is consistently larger for the charged complexes, in agreement with the stronger electrostatic interactions expected in these systems. In contrast, 1-methylimidazolium formate exhibits a distinct interaction profile: the dissociation energy of the ionic complex is notably lower than that of the neutral complex, despite similar equilibrium distances. This suggests a different binding mechanism, likely influenced by the replacement of the aliphatic side chain in the carboxylate with a hydrogen atom. As a result, formate is more polar and substantially smaller than the other anions, which may weaken dispersion and steric contributions to the interaction energy and enhance directional effects.

#### 3.2.2 Limitations of the parabolic approximation and refinement via Morse fitting

Although all data were fitted using Morse potentials, the resulting energy difference $\Delta U_{DA}\left(r_{DA}\right)$ between the charged and neutral complexes, analogous to the $E_{12}$ term in the original Lill and Helms model, can still be extracted and is depicted in Figure 5c. Negative values of $\Delta U_{DA}$ indicate that the product state is energetically more favorable than the reactant. This energy difference exhibits a characteristic dependence on $r_{DA}$ : it decreases initially, reaches a minimum, and subsequently increases with increasing separation, asymptotically approaching a limiting value of $D+D_{0}$ . For formate, the minimum occurs at a donor–acceptor distance below 3.0 Å, while for acetate, propanoate, and butanoate, it lies slightly above this value. The $\Delta U_{DA}\left(r_{DA}\right)$ curves are well-described by Morse potentials in Equation 14 with parameters listed in Table 1, offering a significantly improved fit over the parabolic approximation originally proposed by Lill and Helms (2001a).

**FIGURE 6:**
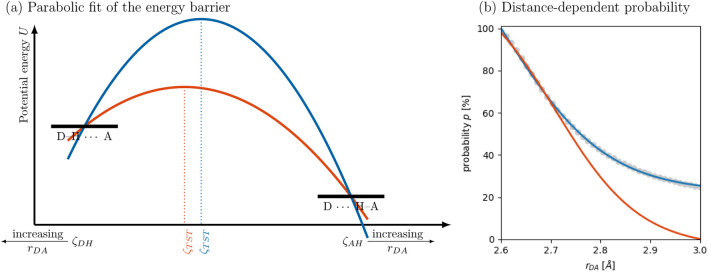
**(a)** Parabolic fit of the proton transfer barrier following the approach of Lill and Helms (2001a). **(b)** The reaction probability based on the kinetic model as a function of the distance $r_{DA}$ .

Furthermore, they assumed that the positions of the two minima at $\zeta_{DH}$ and $\zeta_{AH}$ as well as the energy difference $\Delta U_{DA}$ are sufficient to determine the activation barrier. However, as illustrated in Figure 6a, this assumption is not generally valid: both, the orange and blue parabola contain the reactant and product state but have different energy barriers and positions of the transition state. In the case of proton transfer in water, additional (possibly implicit) physical constraints, such as symmetry, solvent effects, or assumed properties of the transition state, may have allowed Lill and Helms to resolve this ambiguity. One practical way to constrain the fit is to fix the position of the transition state to a specific value of the reaction coordinate $\zeta_{\mathrm{TST}}$ . As will be discussed in later sections, for the PILs studied here, $\zeta_{\mathrm{TST}}$ is consistently found to be close to zero. It is important to note that the positions of the ζ -minima are not symmetric about $\zeta_{\mathrm{TST}}$ , even if $\zeta_{\mathrm{TST}}$ is zero. In other words, $\zeta_{DH}\neq -\zeta_{AH}$ , which is also visible in Figure 6a and can be deduced from the basic definition of the collective variables in Equations 17, 18:

$$\zeta_{DH}=r_{DH}-r_{AH}=2r_{DH}-r_{DA}$$ (17)

$$\zeta_{AH}=r_{DH}-r_{AH}=-2r_{AH}+r_{DA}$$ (18)

One notices that in the reactant state DH⋯A the bond length $r_{DH}$ between the nitrogen of the imidazolium and the hydrogen is 1.01 Å, whereas in the product state D⋯HA the bond length $r_{AH}$ is 0.97 Å. Furthermore, as discussed before and summarized in Table 1, $r_{DA}$ in the reactant state DH⋯A is shorter than in the product state D⋯HA by roughly 0.1 Å.

**FIGURE 7:**
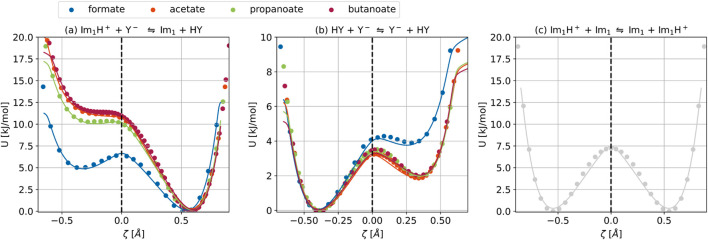
Proton transfer energy profiles for **(a)** the acid–base reaction in Equation 1, **(b)** the side reaction in Equation 8, and **(c)** the reaction in Equation 9, plotted as a function of the collective variable ζ defined in Equation 15. Solid lines represent empirical valence bond (EVB) model fits.

A further limitation of the parabolic fit is its inability to capture cases where no energy barrier exists between the reactant and product states, as observed for the proton transfer reaction ${\text{Im}}_{1}{\text{H}}^{+}+{\text{Y}}^{-}⇌{\text{Im}}_{1}+\text{HY}$ shown in Figure 7a. In such barrierless cases, the parabolic model fails to provide a meaningful description of the transition state. Nonetheless, the energy difference $\Delta U\left(r_{DA}\right)$ between the charged and neutral species, as shown in Figure 5c, can still be exploited to determine the distance-dependent reaction probability used in PROTEX simulations.

#### 3.2.3 Distance-dependent proton transfer probabilities

All molecules that share a hydrogen bond are subject for a proton transfer reaction, when PROTEX interrupts the polarizable MD simulation. Usually, a hydrogen bond is considered if the distance between the donor and acceptor atom is less than 3 Å. Closest distances are around 2.5 Å to 2.6 Å for the reaction of 1-methylimidazolium acetate (Gődény et al., 2024). Taking all acceptors within the first shell of the donor molecule into account, PROTEX overestimates proton hopping (Gődény et al., 2024). Consequently, the proton hopping probabilities should be scaled down with increasing distance. PROTEX currently supports two empirical distance-dependent scaling schemes for this probability: a linear decay and a cosine-based decay, but the current data suggest a new function. In the absence of a well-defined barrier, the difference $\Delta U\left(r_{DA}\right)$ can be used to compute the probability according to the kinetic energy model (Jacobi et al., 2022) in Equation 19:

$$p^{\text{kin}}\left(r_{DA}\right)=\overset{\infty }{\underset{\Delta U\left(r_{DA}\right)}{\int }}\frac{E_{\text{kin}}}{{\left(k_{B}T\right)}^{2}}\;\exp\left[-\frac{E_{\text{kin}}}{k_{B}T}\right]dE_{\text{kin}}$$ (19)

$$=\left(\frac{\Delta U\left(r_{DA}\right)}{k_{B}T}+1\right)\;\exp\left[-\frac{\Delta U\left(r_{DA}\right)}{k_{B}T}\right]$$ (20)

$$≃\min\left(a_{1}\tanh\left[k\left(r_{DA}-r_{DA}^{*}\right)\right]+a_{0},100\%\right)$$ (21)

as a function of the distance $r_{DA}$ . The corresponding probabilities are depicted for butanoate in Figure 6b as gray dots. The resulting probabilities can be approximated by a hyperbolic tangent function in Equation 21. Here, $r_{DA}^{*}$ denotes the turning point of the curve, k defines the steepness of the transition, and the coefficients $a_{1}$ and $a_{0}$ control the amplitude and vertical shift of the probability range. The use of the min function ensures that the calculated probability does not exceed 100% at short donor–acceptor distances. The parameters $a_{1}$ , k and $a_{0}$ in Equation 21 can be determined so that the fit the actual probability data (blue curve in Figure 6b). However, then the probability is not zero at the upper threshold of 3 Å. The alternative fit (orange curve) imposes this constraint, ensuring that proton transfer probabilities are effectively zero for donor–acceptor pairs beyond the defined hydrogen bond threshold. Still, at shorter distances, the exact probabilities are reproduced. The latter approach is preferable, as only pairs within the cutoff are considered eligible for reaction in the PROTEX framework and, consequently, the probability above that threshold is zero. The upcoming version of PROTEX will incorporate this hyperbolic tangent function as a default model for computing distance-dependent proton transfer probabilities, providing a more accurate and flexible description of the distance-dependence.

### 3.3 Analysis of the collective variable concerning the proton transfers

To characterize the proton transfer process, the hydrogen–donor $r_{DH}$ and hydrogen–acceptor $r_{AH}$ distances were individually recorded throughout the QM scans. These values were used to directly compute the collective variable $\zeta =r_{DH}-r_{AH}$ for each energy-minimized configuration during the scan, which provides a continuous descriptor of the proton position along the reaction pathway. As the scans were not performed with fixed increments in ζ but $r_{AH}$ , the resulting reaction profiles presented in Figure 7 exhibit non-uniform spacing in ζ -values. Additionally, the donor–acceptor distance $r_{DA}=r_{DH}+r_{AH}$ was not held constant during the scans and varied slightly as the system relaxed along the proton transfer coordinate. To illustrate this, consider the optimized reactant state for the ionic complex ${\text{Im}}_{1}{\text{H}}^{+}\cdots {\text{Y}}^{-}$ , shown in Figure 5a, which has an equilibrium donor–acceptor distance of $r_{DA}^{0}$ of 2.55 Å. This same configuration appears in Figure 7a at $\zeta_{DH}$ , where the energy profile theoretically should have a minimum (at least visible for the formate). The neutral complex $\text{HY}\cdots {\text{Im}}_{1}$ has the lowest energy in Figure 5b at $r_{DA}^{0}=2.64\text{Å}$ . This configuration is the product state and corresponds to the minimum in Figure 7a located near $\zeta_{AH}\approx 0.57$ .

#### 3.3.1 Comparison of proton transfer reactions across the carboxylate systems

As shown in Figure 7a, the energy profiles for 1-methylimidazolium acetate, propanoate, and butanoate exhibit similar features: a stable neutral product, a less stable ionic reactant, and no visible intrinsic energy barrier for the forward proton transfer. In contrast, the profile for 1-methylimidazolium formate shows a significantly lower-energy reactant state and consequently a small intrinsic reaction barrier of 1.4 kJ mol^−1^. Nevertheless, the neutral species 1-methylimidazole and carboxylic acid are thermodynamically more stable than the ionic forms. Consequently, while the forward proton transfer proceeds without a significant barrier, the back reaction exhibits a substantially higher activation energy, in line with the endergonic nature of reprotonating the imidazole. However, $\Delta U_{\text{AH}}$ of roughly 10 kJ mol^−1^ is relatively small for a reaction barrier, suggesting that the process is kinetically rather than thermodynamically controlled (Qiu and Schreiner, 2023).

The values of the collective variables corresponding to the reactant and product states, $\zeta_{DH}$ and $\zeta_{AH}$ , along with the associated forward and reverse energy barriers, are summarized in Table 2. These quantities provide a detailed characterization of the proton transfer landscape for each system. Based on these energy barriers, the reaction probabilities can be computed using the kinetic energy model described in Equation 20, which accounts for the thermal activation of proton transfer events as a function of the local potential energy difference. Since we included the polarizable continuum model for the QM scans in this study, the barrier and correspondingly the reaction probability slightly differs from the value reported in Reference (Jacobi et al., 2022). It also seems, that the polarizable continuum flattens the barrier.

**TABLE 2:** Key parameter for the proton transfer reactions in Figure 7.

Anion ${\text{Y}}^{-}$	Reactant state			Product state		
	$\zeta_{DH}$	$\Delta U_{DH}$ [kJ/mol]	$p_{DH}^{\text{kin}}$ [%]	$\zeta_{AH}$	$\Delta U_{AH}$ [kJ/mol]	$p_{AH}^{\text{kin}}$ [%]
	${\text{Im}}_{1}{\text{H}}^{+}+{\text{Y}}^{-}$			${\text{Im}}_{1}+\text{HY}$		
Formate	−0.38	1.4	89.1	0.51	6.4	27.4
Acetate	−0.19	< 0.1	100.0	0.58	11.2	6.2
Propanoate	−0.28	0.1	100.0	0.56	10.4	8.0
Butanoate	−0.21	< 0.1	100.0	0.58	11.3	6.0
	$\text{HY}+{\text{Y}}^{-}$			${\text{Y}}^{-}+\text{HY}$		
Formate	−0.39	4.3	48.6	0.29	0.3	99.3
Acetate	−0.40	3.3	61.9	0.34	1.4	89.1
Propanoate	−0.39	3.5	59.1	0.34	1.5	87.8
Butanoate	−0.41	3.5	59.1	0.32	1.5	87.8
​	${\text{Im}}_{1}{\text{H}}^{+}+{\text{Im}}_{1}$			${{\text{Im}}_{1}+\text{Im}}_{1}{\text{H}}^{+}$		
​	−0.55	7.3	21.0	0.55	7.3	21.0

#### 3.3.2 Asymmetry in the symmetric $\text{HY}+{\text{Y}}^{-}⇌{\text{Y}}^{-}+\text{HY}$ proton transfer

**FIGURE 8:**
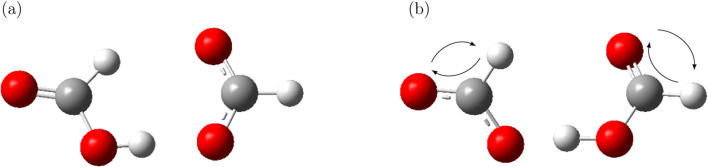
Comparison of the reactant **(a)** and product **(b)** states from the QM scan of formic acid with formate. The structures are not strictly identical, reflecting an asymmetric molecular reorientation. Nevertheless, if the oxygen and hydrogen not involved in the proton transfer switch places in the formate and formic acid, the initial reactant state can be regained. However, this does not involve any change to the collective variable ζ and is consequently not discussed here.

The reaction $\text{HY}+{\text{Y}}^{-}⇌{\text{Y}}^{-}+\text{HY}$ is expected to be symmetric from a theoretical standpoint. However, the energy scan reveals that the reactant and product states are not structurally identical, as illustrated in Figure 8.

In this case, the product state differs from the reactant primarily due to the rotation of the non-hydrogen bonded oxygen and hydrogen of the formic acid and formate. Importantly, this internal reorientation does not alter the key bond lengths associated with the proton transfer coordinate. As a result, the product state in Figure 7b may appear as a second transition-like configuration, though the pathway connecting it back to the reactant state does not involve a proton transfer event or even changing the distance between the proton and the oxygen and is therefore outside the scope of the current analysis. Despite the structural asymmetry, the energy barrier for the reverse reaction (product to reactant) shown in Figure 7b is lower than that for the forward direction. Nevertheless, since the barrier from product to reactant state in Figure 7b is less than the forward reaction, one might argue that in MD simulation a mutual orientation of the product state may readily occur since MD simulations do not care about minimum energies from QM. The proton transfer from this mutual orientation should be easier. This observation suggests that mutual orientation effects could influence the effective proton transfer rate in simulations. Accordingly, the two distinct proton transfer probabilities derived from Table 2 must be explicitly tested within the PROTEX framework to assess their impact on macroscopic properties such as ionic conductivity.

### 3.4 Empirical valence bond analysis

#### 3.4.1 Morse potentials of the individual states

The energy profiles of both reactant and product states as a function of the collective variable ζ can be accurately described using Morse potentials of the form:

$$U\left(\zeta \right)=D{\left(1-\exp\left[-\alpha \left(\zeta -\zeta_{0}\right)\right]\right)}^{2}+D_{0}$$ (22)

Here, D denotes the dissociation energy, $\zeta_{0}$ the equilibrium position of the minimum, and α controls the curvature of the potential. The parameter $D_{0}$ serves as a vertical energy shift to accommodate cases where the energy minimum does not lie at zero, such as in energetically unfavorable states. For instance, in Figure 7a, the reactant state lies higher in energy than the product, while in Figure 7b, the product state is the less favorable configuration. Although $D_{0}$ is only required for the unfavorable states, it was uniformly included in all fits for consistency. To model the product state, which lies on the right side of the energy profile (positive ζ ), a negative value of α is used to reflect the inversion of the potential about its minimum. Furthermore, to determine the value of $\zeta_{DH}$ , the location of the reactant minimum, the Morse potential was fitted only over the domain $\zeta <\zeta_{DH}$ in Figure 7a. The extracted $\zeta_{DH}$ values are reported in Table 2 and provide a reliable estimate of its position in the absence of a transition state barrier.

#### 3.4.2 Coupling of reactant and product states

The complete reaction profile consisting of these individual states can be determined by Empirical Valence Bond (EVB) theory (Gődény and Schröder, 2025; Oanca et al., 2023; Warshel and Weiss, 1980; Åqvist and Warshel, 1993), which provides a computationally efficient framework for simulating chemical reactions, particularly those involving proton or electron transfer, in condensed-phase environments (Day et al., 2002; Knight et al., 2012; Voth, 2006; Čuma et al., 2000). It offers a balance between QM accuracy and efficiency for large molecular systems and long time scales, making it well-suited for modeling reactive processes in complex media.

EVB represents the potential energy surface of a reaction as a combination of diabatic (non-interacting) states, typically corresponding to reactant and product configurations. The adiabatic energy is obtained by diagonalizing the EVB Hamiltonian, which includes off-diagonal coupling elements to account for QM mixing. While multi-state EVB models have been successfully applied to proton transfer in water (Chen et al., 2010; Schmitt and Voth, 1998; Wu et al., 2008), the present work employs a simplified two-state formalism along the reaction coordinate ζ , which characterizes the proton transfer process considered here. The EVB coupling between the reactant and product states is expressed by the following 2×2 determinant in Equation 23:

$$\begin{array}{ccc} \left|\begin{array}{cc} U_{DH}\left(\zeta \right)-E\left(\zeta \right) & E_{DA}\\ E_{DA} & U_{AH}\left(\zeta \right)-E\left(\zeta \right) \end{array}\right| & = & 0 \end{array}$$ (23)

$$E\left(\zeta \right)=\frac{1}{2}\left(U_{DH}\left(\zeta \right)+U_{AH}\left(\zeta \right)\right)-\sqrt{{\left(U_{DH}\left(\zeta \right)-U_{AH}\left(\zeta \right)\right)}^{2}+4\;E_{DA}^{2}}$$ (24)

with a constant coupling term $E_{DA}$ of the EVB model. Traditionally, EVB models employ harmonic potentials to represent the individual diabatic states. In contrast, the present approach introduces Morse potentials, $U_{DH}\left(\zeta \right)$ and $U_{AH}\left(\zeta \right)$ , as defined in Equation 22, thereby incorporating anharmonic effects even in the vicinity of $\zeta_{DH}$ and $\zeta_{AH}$ . To the best of our knowledge, this represents the first application of Morse-type EVB potentials for describing proton transfer in such systems. The resulting adiabatic energy E(ζ) profiles are depicted for our systems under investigation as solid lines in Figure 7. The corresponding parameters are given in Table 3. The mean absolute error for E(ζ) is lower than 0.4 kJ mol^−1^ for all reactions, when restricting ζ between −0.6 and 0.6. The Morse parameters were optimized to match the relaxed potential energy scans, and thus the precise values may differ slightly from those directly extracted from the unprocessed data in Table 2.

**TABLE 3:** Empirical valence bond fit parameters for the reaction profiles.

Anion ${\text{Y}}^{-}$	Reactant state				Product state				
	D [kJ/mol]	α [Å^−1^]	$\zeta_{0}$ [Å]	$U_{0}$ [kJ/mol]	D [kJ/mol]	α [Å^−1^]	$\zeta_{0}$ [Å]	$U_{0}$ [kJ/mol]	*E_DA_* [kJ/mol]
	${\text{Im}}_{1}{\text{H}}^{+}+{\text{Y}}^{-}⇌{\text{Im}}_{1}+\text{HY}$								​
Formate	9.5	2.01	−0.32	4.9	12.3	−2.59	0.55	0.2	0.5
Acetate	0.2	3.61	−0.06	11.0	23.6	−2.02	0.59	0.3	0.5
Propanoate	2.7	2.50	−0.22	10.1	19.3	−2.30	0.59	0.2	0.5
Butanoate	1.8	2.52	−0.16	11.2	20.4	−2.28	0.60	0.2	0.5
	$\text{HY}+{\text{Y}}^{-}⇌{\text{Y}}^{-}+\text{HY}$								​
Formate	11.6	2.36	−0.40	0.1	3.4	−2.60	0.26	3.8	0.5
Acetate	9.8	2.44	−0.38	0.1	5.2	−2.61	0.33	1.9	0.5
Propanoate	9.7	2.52	−0.39	0.1	4.1	−3.15	0.35	2.0	0.5
Butanoate	9.2	2.55	−0.39	0.1	3.3	−3.47	0.35	2.1	0.5
​	${\text{Im}}_{1}{\text{H}}^{+}+{\text{Im}}_{1}⇌{\text{Im}}_{1}+{\text{Im}}_{1}{\text{H}}^{+}$								​
​	16.1	2.18	−0.55	0.4	16.1	−2.18	0.55	0.4	0.7

The mean absolute error between the EVB, and the QM, potential is less for 0.4 kJ mol^−1^ for all reactions.

The coupling energies $E_{DA}$ are found to be consistently small, with values below 1 kJ mol^−1^. These couplings are significantly smaller than the thermal energy at room temperature ( $k_{B}T≃$ 2.5 kJ mol^−1^) and two orders of magnitude lower than those reported for proton transfer in aqueous systems (Wu et al., 2008). The comparatively low coupling energies may arise from the use of Morse potentials rather than harmonic potentials, as the former exhibit a more gradual energy increase towards dissociation. Consequently, $E_{DA}$ in Equation 24 can be smaller for almost flat potential energy surfaces compared to employing harmonic potentials for reactant and product state. Except for 1-methylimidazolium formate, the proton-transfer reactions shown in Figure 7a exhibit a flat potential energy surface with no discernible energy barrier. As noted above, the donor–acceptor distance $r_{DA}^{0}$ between the donor nitrogen and the acceptor oxygen is below 2.6 Å for the ${\text{Im}}_{1}{\text{H}}^{+}+{\text{Y}}^{-}$ complex, placing these systems within the regime of low-barrier hydrogen bonds as defined by Ishikita and Saito (2014). A characteristic feature of these reactions is the position of the transferring hydrogen, which resides essentially midway between the donor and acceptor atoms at the transition state. This is consistent with our observation that most transition states occur at $\zeta_{\mathrm{TST}}≃0$ . Although still a energy gap between reactant and product state in our investigated systems exist, the narrow intrinsic barrier width forces the potentials of the reactant and product states into close proximity. This leads to an adiabatic crossing with low energy barriers (see Figure 1 of Ref. Ishikita and Saito, 2014) and, consequently, to the markedly small coupling energies $E_{DA}$ observed in our systems. This peculiar behavior cannot be extrapolated to other PILs without further QM computations.

Despite the flat potential energy surfaces, substantial populations of the charged species ${\text{Im}}_{1}{\text{H}}^{+}$ and ${\text{Y}}^{-}$ must be present in PILs, as evidenced by their appreciable ionic conductivities. This indicates that the ions behave as entropically trapped intermediates (Meot-Ner and Smith, 1991; Yang et al., 2019), implying that proton transfer in these systems is not primarily governed by enthalpic contributions. Meot-Ner and Smith (1991) postulated several steps of the reaction pathway of these entropically trapped intermediates: First, a loose collision complex forms. Second, efficient proton transfer, however, requires a highly specific mutual orientation of the reacting partners. As internal rotations become restricted, the complex correspondingly tightens, and entropy becomes the dominant factor controlling this stage of the process. Third, once a strong hydrogen bond is established, the proton can transfer from donor to acceptor. This transfer proceeds notably slower than would be expected on the basis of the potential energy surface alone (Meot-Ner and Smith, 1991).

### 3.5 Quantum effects

This behavior in our systems suggest that proton transfer occurs primarily through thermally activated transitions rather than via QM delocalization or tunneling. The low coupling reflects the limited mixing of reactant and product states at the transition state (typically near $\zeta_{\mathrm{TST}}$ ), where neither diabatic state has reached its dissociation limit. This finding also supports the applicability and success of the kinetic energy model previously employed to estimate reaction probabilities (Gődény and Schröder, 2025; Jacobi et al., 2022), as the system behavior is dominated by classical over-the-barrier transitions rather than quantum coherence effects (Takatsuka et al., 1999).

#### 3.5.1 Wentzel-Kramers Brillouin tunneling

To quantify quantum nuclear effects in the proton-transfer reactions studied here, we evaluated the one-dimensional Wentzel–Kramers–Brillouin (WKB) (Griffiths, 2012; Takatsuka et al., 1999; Wentzel, 1926) transmission probability $p^{\text{WKB}}$ along the intrinsic reaction coordinate ζ . Assuming that motion perpendicular to ζ is separable and that the adiabatic ground-state potential U(ζ) is sufficiently smooth, the probability in Equation 25 for a proton of mass μ to tunnel from the reactant well to the product well is:

$$\ln p^{\text{WKB}}=-\frac{2}{\hbar }\int \sqrt{2\mu \left(U\left(\zeta \right)-E_{\text{thermal}}^{\text{QM}}\right)}\;d\zeta$$ (25)

where the lower integration limit is the ζ at which $U\left(\zeta \right)=E_{\text{thermal}}^{\text{QM}}$ and the upper limit corresponds to the equilibrium ζ of the respective product state. The proton does not tunnel from the absolute vibrational ground state but it has a finite amount of thermal energy in Equation 26 available from its vibrational mode. In the harmonic quantum limit that energy is:

$$E_{\text{thermal}}^{\text{QM}}\left(\nu \right)=\frac{h\nu }{\exp\left[\frac{h\nu }{k_{B}T}\right]-1}$$ (26)

$$\nu ≃\frac{1}{2\pi }\sqrt{\frac{2\alpha^{2}\cdot D}{\mu }}$$ (27)

using the harmonic frequency ν in Equation 27, which can be estimated from the Morse potentials in Table 3. The corresponding frequencies and thermal energies are summarized in Table 4.

**TABLE 4:** Contribution of quantum tunneling of the proton based on Wentzel, Kramers and Brillouin (WKB) approximation.

Anion ${\text{Y}}^{-}$	$\nu {[\text{cm}}^{-1}$ ]	$E_{\text{thermal}}^{\text{QM}}$ [kJ/mol]	$p^{\text{WKB}}$ [%]	$p^{\text{kin}}$ [%]	P [%]
	${\text{Im}}_{1}+\text{HY}⇌{\text{Im}}_{1}{\text{H}}^{+}+{\text{Y}}^{-}$				
Formate	677	0.33	0.1	27.4	27.5
Acetate	733	0.27	0.3	6.2	6.4
Propanoate	754	0.25	0.1	8.0	8.1
Butanoate	769	0.24	0.1	6.0	6.1
	$\text{HY}+{\text{Y}}^{-}⇌{\text{Y}}^{-}+\text{HY}$				
Formate	600	0.43	1.5	48.6	49.3
Acetate	570	0.48	2.9	61.9	63.0
Propanoate	586	0.45	2.2	59.1	60.0
Butanoate	578	0.46	2.6	59.1	60.1
​	${\text{Im}}_{1}{\text{H}}^{+}+{\text{Im}}_{1}⇌{\text{Im}}_{1}{\text{H}}^{+}+{\text{Im}}_{1}$				
​	653	0.36	0.1	21.0	21.1

The obtained thermal energies are significantly lower than the classical thermal limit of 2.5 kJ mol^−1^ because only the lowest vibrational modes are occupied at a temperature of 300 K.

The intrinsic barrier width (Qiu and Schreiner, 2023) (see Figure 3b) serves as a measure of the tunneling distance. In the reaction ${\text{Im}}_{1}{\text{H}}^{+}+{\text{Y}}^{-}⇌{\text{Im}}_{1}+\text{HY}$ , however, the energy barrier is negligibly small for ${\text{Y}}^{-}$ ∈ {acetate, propanoate, butanoate}, resulting in a very flat transition state. Consequently, the intrinsic width cannot be meaningfully defined in these cases. In contrast, for the side reactions shown in Figures 7b,c, the intrinsic widths are approximately 0.5 Å and 1 Å, respectively. Accordingly, the tunneling probability for $\text{HY}+{\text{Y}}^{-}⇌{\text{Y}}^{-}+\text{HY}$ is expected to be considerably higher than that for ${\text{Im}}_{1}{\text{H}}^{+}+{\text{Im}}_{1}⇌\;{\text{Im}}_{1}+{\text{Im}}_{1}{\text{H}}^{+}$ , which is case for our WKB probabilities in Table 4.

#### 3.5.2 Contribution to the proton reaction probabilities

Although the WKB model neglects multidimensional effects and assumes a slowly varying barrier, it offers a numerically inexpensive upper bound on tunneling contributions, well suited to the present screening study. Across all systems in Table 4, $p^{\text{WKB}}$ is at least one order of magnitude smaller than the classical thermal probability $p^{\text{kin}}$ , confirming that classical over-the-barrier hopping dominates proton transfer in these PILs. Nonetheless, $p^{\text{WKB}}$ is part of the overall probability p in Equations 28, 29:

$$p=\left(1-p^{\text{WKB}}\right)\cdot p^{\text{kin}}+p^{\text{WKB}}$$ (28)

$$=\left(1-p^{\text{kin}}\right)\cdot p^{\text{WKB}}+p^{\text{kin}}$$ (29)

to account for the rare events where tunneling bypasses the classical pathway. Because $p^{\text{kin}}\gg p^{\text{WKB}}$ in every case, Equation 29 reduces numerically to $p≃p^{\text{kin}}$ . We therefore conclude that explicit inclusion of quantum tunneling would not alter the mechanistic picture put forward in this study. Finally, we note that $p^{\text{WKB}}$ is highly sensitive to subtle changes in barrier height and width. Consequently, future studies employing higher-level electronic-structure methods or path-integral simulations may observe modest quantitative shifts in the values reported here, although the qualitative conclusion, that tunneling is a minor contributor, should remain robust.

## 4 Conclusion and outlook

In this work, we investigated the proton transfer mechanism in a series of 1-methylimidazolium-based protic ionic liquids through quantum-mechanical potential energy scans along two collective variables: the donor–acceptor distance $r_{\text{DA}}=r_{\text{DH}}+r_{\text{AH}}$ and the proton transfer coordinate $\zeta =r_{\text{DH}}-r_{\text{AH}}$ as illustrated in Figure 2. The resulting energy landscapes reveal that proton migration proceeds via a nearly one-dimensional pathway characterized by asymmetric potential profiles that energetically favor the formation of neutral species. Fitting reactant and product state with Morse potentials proved superior to previously used parabolic approximations. Since the Empirical Valence Bond coupling between the reactant and product states ranged from 0.5 kJ mol^−1^ to 0.7 kJ mol^−1^, which is considerably smaller than the thermal energy at ambient temperature, the corresponding proton transfer processes proceed predominantly via thermally activated hops, as captured by our kinetic model. The calculated reaction probabilities are accordingly much higher than those predicted by quantum tunneling. With the exception of 1-methylimidazolium formate, the investigated carboxylate systems exhibit nearly identical probabilities for all forward and backward proton transfer reactions hinting at the negligible influence of the alkyl chain on the proton transfer for these PILs.

**FIGURE 9:**
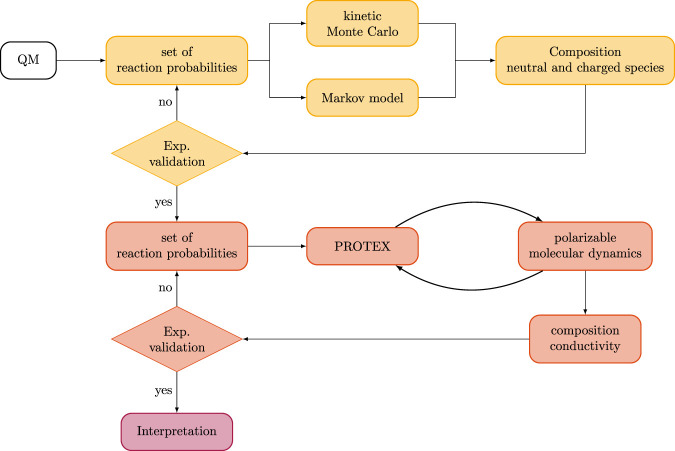
Workflow of the parametrization of the reaction probabilities when using PROTEX. The yellow loop represents the validation of reaction probabilities based on statistical models that operate solely on molecular concentrations, without accounting for attractive or repulsive interactions between species. The orange loop illustrates the integration of PROTEX with a molecular dynamics program to perform reactive simulations. In this stage, the PROTEX parameters include not only the reaction probabilities but also the interval between protonation events and the distance dependence of these probabilities.

As illustrated in Figure 9, the complete set of reaction probabilities obtained from the quantum-mechanical calculations feeds into a subsequent validation cycle (yellow boxes). Within this framework, the overall composition of the protic ionic liquid will be evaluated using kinetic Monte Carlo simulations (Andersen et al., 2019; Voter et al., 2007) or Markov models (Jacobi et al., 2022). In contrast to the quantum-mechanical approach, these stochastic models explicitly account for the competition among concurrent reactions as well as for the temporal depletion or accumulation of specific molecular species. The computed equilibrium concentrations will be validated against the experimentally determined composition. Compositions obtained through this kinetic modeling approach are expected to be considerably more reliable than those inferred directly from the thermodynamic quantities, such as $\Delta G_{\text{IL}}$ , presented in this work. For 1-methylimidazolium acetate, the quantum-mechanical reaction probabilities previously yielded a realistic composition when evaluated within a Markov framework (Jacobi et al., 2022). As the probabilities obtained in the present study closely match the earlier values, a similarly good agreement with experimental data is anticipated.

In the subsequent stage of the workflow (orange boxes in Figure 9), the set of reaction probabilities is incorporated into the PROTEX framework, where it is combined with distance-based criteria to identify hydrogen-bonded reactive pairs. At present, PROTEX employs either a fixed distance cut-off or a linear function to describe the distance dependence of the reaction probability. The present quantum-mechanical scans along the collective variable $r_{\text{DA}}$ reveal a more realistic distance dependence that can be accurately represented by a hyperbolic tangent function, which will be implemented in future versions of PROTEX.

Reactive molecular dynamics simulations performed with PROTEX further account for diffusion, mutual electrostatic interactions, and steric effects among the various species—phenomena that are not captured by Markov state models or kinetic Monte Carlo approaches. Ultimately, the simulations will be validated not only by comparing the predicted equilibrium composition with experimental data but also by evaluating the ionic conductivity, which explicitly includes contributions from proton hopping. Because conductivity is directly measurable, unlike composition which is often inferred indirectly, agreement with experimental conductivities provides a particularly stringent and meaningful test of the overall model. Successful reproduction of these observables would confirm the consistency and predictive capability of the multiscale framework, originating from the quantum-mechanical determination of individual reaction probabilities.

## Data Availability

The raw data supporting the conclusions of this article will be made available by the authors, without undue reservation.
